# Type VI secretion system activity at lethal antibiotic concentrations leads to overestimation of weapon potency

**DOI:** 10.1099/mic.0.001600

**Published:** 2025-08-21

**Authors:** William P. J. Smith, Elisa T. Granato

**Affiliations:** 1 Division of Evolution, Infection and Genomics, Faculty of Biology, Medicine and Health, University of Manchester, Manchester, UK; 2 Department of Biology, University of Oxford, Oxford, UK

**Keywords:** competition assays, interbacterial competition, microbe–microbe interactions, type VI secretion systems

## Abstract

Competition assays are a mainstay of modern microbiology, offering a simple and cost-effective means to quantify microbe–microbe interactions *in vitro*. Here, we demonstrate a key weakness of this method that arises when competing microbes interact via toxins, such as those secreted via the type VI secretion system (T6SS). Time-lapse microscopy reveals that T6SS-armed *Acinetobacter baylyi* bacteria can maintain lethal T6SS activity against *E. coli* target cells, even under selective conditions intended to eliminate *A. baylyi*. Further, this residual killing creates a density- and T6SS-dependent bias in the apparent recovery of *E. coli*, leading to a misreporting of competition outcomes where target survival is low. We also show that incubating *A. baylyi/E. coli* co-cultures in liquid antibiotic prior to selective plating can substantially correct this bias. Our findings demonstrate the need for caution when using selective plating as part of T6SS competition assays, or assays involving other toxin-producing bacteria.

## Introduction

‘Cut off a wolf’s head and it still has the power to bite’

**Hayao Miyazaki, もののけ姫 [Princess Mononoke: The First Story]**

Practised for over a century, colony-forming unit (c.f.u.) counting is a fundamental microbiological technique for determining the density of bacterial cells in a sample [1]. A common application for this technique is in profiling the potencies of bacterial anti-competitor toxins, as part of a killing assay [2]. Here, a ‘susceptible’ target strain is mixed with a weapon-bearing ‘attacker’ strain. After a set incubation period, during which the attacker kills the susceptible strain, the strain mixture is recovered, diluted and transferred to selective media for c.f.u. counting. The number of susceptible cells counted on media selective for that cell type is then used as a quantitative estimate of weapon potency and/or weapon susceptibility [3,4].

A key assumption of this workflow is that killing due to toxin secretion occurs primarily during the co-culture phase, rapidly ceasing once strain mixtures are transferred to selective media. However, some anti-competitor killing mechanisms operate on fast timescales relative to the bactericidal or bacteriostatic effects of the selective media. These fast-killing mechanisms include the type VI secretion system (T6SS), a versatile anti-competitor weapon found in a broad range of gram-negative bacteria [5,6]. Resembling a poison-tipped speargun [6], the T6SS can inject toxic effector proteins directly into neighbouring target cells, resulting in rapid (<10 min) lysis [7].

These observations prompted us to ask: when using c.f.u. counting in a T6SS killing assay, can interbacterial antagonism continue despite lethal selection on antibiotic-containing media? Does this influence the apparent outcome of the competition? To address these questions, we used fluorescence microscopy to measure the extent and impact of T6SS activity in *Acinetobacter baylyi* cells grown on selective media. We found that, surprisingly, T6SS activity was not detectably reduced on short (10–20 min) timescales during lethal selection. This residual activity proved sufficient to kill the majority of co-plated susceptible *Escherichia coli* cells within minutes.

We then devised a simple ‘ground-truth’ experiment, mixing T6SS attacker and susceptible strains in known ratios and plating these on selective media to mimic the workflow of a typical killing assay. This showed that unwanted post-selection killing introduces significant biases in assay outcomes, particularly where attackers greatly outnumber susceptible cells. Biases are increased the longer cells spend in suspension before plating but are substantially mitigated if an antibiotic pretreatment is used. Our results demonstrate that post-selection killing via contact-dependent weaponry can introduce significant biases in killing assays, leading to overestimation of weapon potency or underestimation of resistance. Based on these findings, we recommend a cautious approach when conducting and interpreting c.f.u.-based killing assays.

## Methods

### Bacterial strains and culture conditions 

The bacterial strains used in this study are listed in Table 1. All strains were routinely cultured from frozen stocks (25 % v/v glycerol) in 5 ml Lysogeny Broth (LB; per litre MilliQ water: 10 g tryptone, 10 g NaCl and 5 g yeast extract) supplemented with antibiotics (50 µg ml^−1^ kanamycin sulphate for *E. coli*, 50 µg ml^−1^ streptomycin sulphate for *A. baylyi* strains) in 50 ml polypropylene conical tubes. Strains were routinely pre-cultured overnight in shaking incubators (180 r.p.m., 16–18 h) at 37 °C (*E. coli*) or at 30 °C (*A. baylyi*), with lids taped 1/4 turn loose to allow aeration. Optical density of liquid cultures was measured at 600 nm (OD_600_). For c.f.u. counting, we used 120 mm vented square plates filled with ~40 ml molten LB agar (1.5 % w/v), supplemented with antibiotics where necessary, as noted below. For time-lapse microscopy experiments, samples were kept at 30 °C throughout the duration of the experiment using a custom-built incubation chamber.

**Table 1.** Bacterial strains used in this study

Label	Species	Genotype	Source
MG1655	*E. coli*	MG1655::EGFP Kan^R^	[31]
WT	*A. baylyi*	ADP1 *rpsL-K88R clpV-mCherry2* Strep^R^	Marek Basler
*∆tssM*	*A. baylyi*	ADP1 *rpsL-K88R* ∆*tssM clpV-mCherry2* Strep^R^	Marek Basler
WT-*gfp*	*A. baylyi*	ADP1 *rpsL-K88R vipA-sfGFP clpV-mCherry2* Strep^R^	[3]
∆*hcp-gfp*	*A. baylyi*	ADP1 *rpsL-K88R* ∆*hcp vipA-sfGFP clpV-mCherry2* Strep^R^	[3]

### Microscopy

#### Precultures

**Fig. 1.**
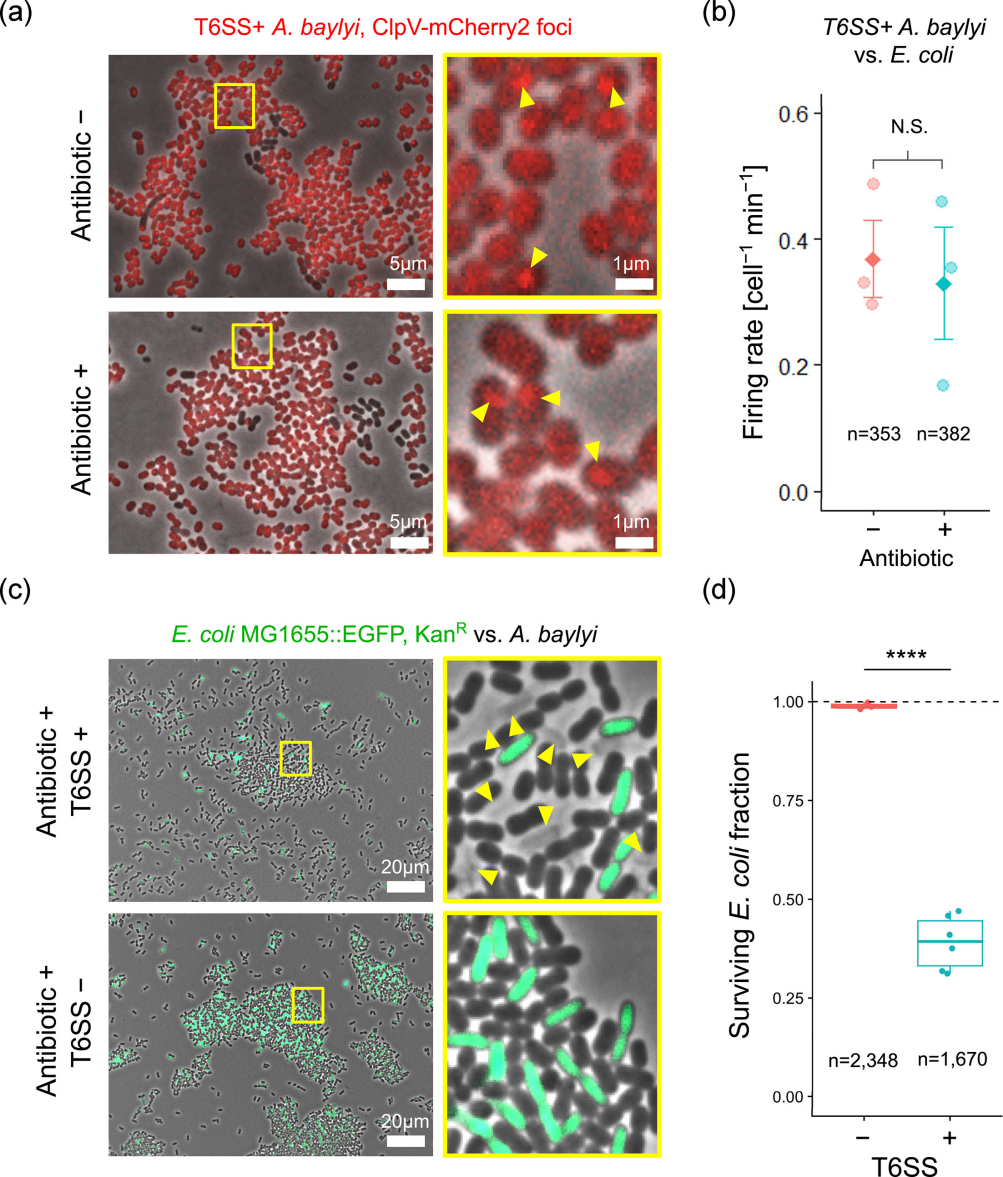
*A. baylyi* T6SS firing and *E. coli* killing are transiently maintained under lethal antibiotic selection. (**a**) Top row: overnight culture of T6SS+ *A. baylyi* (‘WT’: ADP1 *rpsL-K88R clpV-mCherry2* Strep^R^) bacteria growing on LB agar; the yellow box in the left panel corresponds to the zoomed section on the right. mCherry2-tagged ClpV proteins (red; arrows) are visible as dynamic foci. Bottom row: as top row, but under lethal selection (100 µg ml^−1^ kanamycin). (**b**) Quantification of T6SS firing rate (counting ClpV foci) in exponential phase T6SS+ WT *A*. *baylyi* in 1 : 1 co-culture with *E. coli* MG1655, 10–20 min after spotting on LB agar, based on 18 images across 3 independent experiments. N.S.: non-significant (Wilcoxon rank sum test, *n*=3, *W*=5; *P*=1.0). (**c**) Top row: T6SS+ *A. baylyi* WT in 1 : 1 co-culture with EGFP-tagged *E. coli* MG1655 bacteria (green). The yellow box in the left panel corresponds to the zoomed section on the right. The outlines of lysed *E. coli* cells are visible, enabling quantification of killing within a 10-min timeframe. Bottom row: as top row but with T6SS− *A. baylyi ∆tssM*. (**d**) Measurements of surviving *E. coli* fraction (live cells/(live+dead cells)) within a 10-min timeframe for T6SS− and T6SS+ *A. baylyi* co-cultures (Welch’s two-sample one-sided t-test, *t*=−21.26, df=5.34, ****P-value<0.0001).

For images shown in Fig. 1(a), overnight cultures of *A. baylyi* WT (T6SS+) were washed twice with LB, and 1 ml of washed culture was resuspended in 50 µl LB. For data and images shown in Fig. 1(b–d), *A*. *baylyi* WT (T6SS+), *ΔtssM* (T6SS−) and *E. coli* cells were diluted into fresh LB medium from overnight cultures, grown to exponential phase (OD_600_ ~0.6–1.0), washed twice with LB medium and resuspended and normalized in LB medium to an OD_600_ of 1.0 (Fig. 1b) or 2.0 (Fig. 1c, d).

#### Sample preparation

*E. coli* was mixed with *A. baylyi* WT (T6SS+) at a ratio of 1 : 1 (20 µl each; Fig. 1b) or with either WT or *ΔtssM* (T6SS−) at a ratio of 5 : 1 (100 µl *A*. *baylyi *+ 20 µl *E. coli*; Fig. 1c, d). For each strain or strain mixture, 1 µl of cell suspension was then spotted onto a 1 % w/v LB agarose pad, supplemented with 100 µg ml^−1^ kanamycin when appropriate. Pads were left to dry for 5 min at room temperature and then inverted onto a 5 cm diameter glass bottom Petri dish with a 3 cm diameter uncoated n° 1.5 glass window (MatTek Corporation), so that the cells were sandwiched between the agarose and the glass. Samples were moved to the microscope and imaged immediately.

#### Image acquisition

Fluorescence microscopy was performed using a Zeiss Axio Observer inverted microscope with a Zeiss Plan-Apochromat 63× oil immersion objective (NA=1.4) and ZEN Blue software (version 1.1.2.0). Image acquisition began ~10 min after spotting on agarose pads. Images were recorded for 15 min at 10-s intervals (Fig. 1a), 5 min at 10-s intervals (Fig. 1b) or just once (Fig. 1c, d). Exposure times were 30–80 ms for phase contrast, 20 ms for EGFP (ex: 488 nm | em: 507 nm) and either 100 ms (Fig. 1a) or 500 ms (Fig. 1b) for mCherry2 (ex: 589 nm | em: 610 nm).

#### Image analysis – firing rate

We measured the rate of T6SS firing in a total of *N*=735 *A*. *baylyi* cells (Fig. 1b) across three independent experiments conducted on different days, via detection of ClpV (mCherry2) fluorescent foci [7]. Each new ClpV focus was assumed to mark one T6SS contraction event in a focal cell. For each experiment, three separate fields of view at different locations on the same agarose pad were monitored for each treatment, yielding a total of 18 time-lapse image series. Image analysis was performed using FIJI 2.1.0/1.53c [8]. For each field of view, total numbers of cells and ClpV foci were determined each minute for 3 min via thresholding, segmentation and object counting in the phase contrast channel and the FIJI built-in ‘Find maxima’ function in the mCherry2 fluorescence channel, respectively.

#### Image analysis – live vs. dead

To enumerate live and dead *E. coli* cells (Fig. 1d), we analysed nine fields of view taken from an LB agarose pad either with kanamycin supplementation (*n*=6) or without (*n*=3). We divided each ~200×160 µm field of view into 16 zones of size ~50×40 µm. *E. coli* densities within each zone varied from 0 to ~150 cells. Green cells were assumed to be live *E. coli*; the outlines of lysed cells lacking EGFP signal were assumed to be dead *E. coli* (see Fig. 1c for examples). Cells within zones in which either (i) focus was too poor to reliably distinguish dead cell outlines or (ii) *E. coli* EGFP fluorescence was too weak to be reliably differentiated from *A. baylyi* cells were ignored (20/144 fields). Dead cells were almost completely absent in the T6SS− samples and were almost exclusively found in direct contact with morphologically distinct *A. baylyi* cells in the T6SS+ samples, confirming that these dead cells are the product of T6SS-mediated killing, and not some other lethal process (e.g. antibiotic selection). Live *E. coli* cell fractions were then calculated as the ratio of live: (live+dead) *E. coli* cells. We compared the measurements from T6SS+ and T6SS− treatments using Welch’s two-sample one-sided t-test (built-in *t.test* method, R [9] version 4.3.2 (2023-10-31)).

### Ground-truth experiment

#### Preparation of cell mixtures

Fifty microlitres of *E. coli* and 500 µl of *A. baylyi* WT-*gfp* (T6SS+) and *Δhcp-gfp* (T6SS−) overnight cultures were transferred into 5 ml sterile LB (respectively, 1 : 100 and 1 : 10 dilution). These cultures were supplemented with the same antibiotics as above and incubated for a further 2.5 h to give a final OD_600_ of ~1.4. Then, 1.5 ml of each exponential phase culture was washed twice with 1 ml Dulbecco’s PBS (dPBS; 20,000 ***g*** centrifugation for 4 min). Each washed culture was then adjusted to OD_600_=0.25 by diluting with dPBS, before being subjected to 5×10 fold serial dilutions (100 µl:900 µl) in dPBS. The *E. coli* density within each of these dilutions was measured independently by plating in sextuplet on pre-dried, pre-warmed non-selective LB agar plates (5 µl per droplet). Once these droplets had dried, c.f.u. plates were lidded, inverted and incubated overnight at 37 °C until individual c.f.u. values could be counted. After sampling for ground-truth counts, 200 µl of washed, normalized cultures of WT-*gfp* or *Δhcp-gfp* were individually mixed with 200 µl of each *E. coli* dilution and then vortexed for 5 s, giving 1 : 1, 10 : 1, 100 : 1, 1,000 : 1, 10,000 : 1 and 100,000 : 1 *A*. *baylyi/E. coli* mixes for each *A. baylyi* treatment. In the top row of a 96-well microtiter plate, 150 µl of each mixture was then combined with either 150 µl dPBS or 150 µl dPBS+50 µg ml^−1^ kanamycin. Each plate was wrapped in Parafilm and incubated at room temperature (~20 °C).

#### C.f.u. counting

After incubating plates for either 45 min, 1 h 45 min, 2 h 45 min or 20 h (overnight), we counted the apparent *E. coli* and * A. baylyi* c.f.u. density in each mixture, plating an independent dilution series for each mixture and time point (7×10 fold serial dilutions in dPBS or dPBS+50 µg ml^−1^ kanamycin; 5 µl of each dilution plated in triplicate on pre-dried, pre-warmed selective LB agar). To measure strain recovery, we used LB agar supplemented with 50 µg ml^−1^ kanamycin (for *E. coli*) or 100 µg ml^−1^ streptomycin (for *A. baylyi*, shown in Fig. S1, available in the online Supplementary Material). Once droplets had dried, selection plates were lidded, inverted and incubated overnight at 37 °C (for *E. coli*) or 30 °C (for *A. baylyi*) until individual colonies could be counted (~12–18 h).

#### Statistical analysis

To compare *E. coli* c.f.u. values in ground-truth, T6SS+ and T6SS− treatments, we fitted a linear mixed effects model using R’s [9] *nlme* package (max. log likelihood fitting with attacker strain, pretreatment, dilution and dilution squared as fixed effects; plating replicate as random effect), at each experimental timepoint. We then used ANOVA to compare fixed effect sizes and their interactions and significances within each fitted model.

## Results

### *A. baylyi* attackers maintain T6SS firing during lethal antibiotic selection

*A. baylyi* is a model organism often used to study T6SS weapon function [3,7, 10,13]. We wanted to test whether *A. baylyi* could still fire its T6SS apparatus under lethal selection conditions and so used single-cell microscopy to image *A. baylyi* cells labelled with a ClpV-mCherry2 fluorescent tag. The protein ClpV is an essential component of the T6SS, and ClpV foci can provide dynamic information on the rate of T6SS firing [3,7, 13]. Overnight cultures of *A. baylyi* WT showed dynamic ClpV foci in both the absence and presence of lethal anti-*A. baylyi* selection (0 or 100 µg ml^−1^ kanamycin), indicating residual T6SS activity under selective conditions (Fig. 1a). To quantify changes in T6SS firing rate under the conditions of a typical competition assay, we repeated this experiment using a 1 : 1 mixture of exponential phase *A. baylyi* WT and *E. coli* cells, individually normalized to OD_600_=1. By counting ClpV foci per *A. baylyi* cell (Fig. 1b) over time, we enumerated T6SS firing events in *A. baylyi* cells grown in co-culture with *E. coli* in the absence and presence of lethal anti-*A. baylyi* antibiotic selection (0 or 100 µg ml^−1^ kanamycin). Surprisingly, we found that, ~10–20 min post-exposure to the antibiotic, T6SS firing rate showed no significant change compared with an antibiotic-free control (Fig. 1b; Wilcoxon rank sum test, *P*=1.0). This demonstrates that, on short timescales, T6SS activity is maintained even under lethal antibiotic selection.

### Residual T6SS activity kills *E. coli *on selective media 

Next, we wanted to test whether post-selection T6SS activity could kill a susceptible competitor strain, under antibiotic conditions intended to exclude the T6SS attacker. Imaging cocultures ~10–15 min post-mixing, we observed extensive killing of * E. coli* cells, evident from rapid lysis and concurrent loss of EGFP signal (Fig. 1c) [3]. After lysis, *E. coli* cells remained visible as faint silhouettes, corresponding to the remnants of compromised cell envelopes [3]. By counting the number of intact and lysed * E. coli* cells across multiple views of the same co-culture sample, we estimated the extent of T6SS-mediated killing. Under selective conditions lethal to *A. baylyi* (100 µg ml^−1^ kanamycin), we observed that >50% of *E. coli* cells died after 10–15 min of coculture with T6SS+ *A. baylyi* attackers (Fig. 1d). By contrast, lysis rates were minimal in a T6SS− control (Fig. 1d), indicating that *E. coli* killing was indeed the product of T6SS activity. This rapid killing is consistent with previous observations of T6SS dynamics in *A. baylyi* and other species [3,7, 14, 15]. Crucially, it shows that significant T6SS killing is possible even under antibiotic selection conditions that are lethal to the attacker strain*,* highlighting the potential for induced bias in killing assay outcomes.

### Post-selection killing creates density-dependent bias in apparent competition outcomes 

**Fig. 2.**
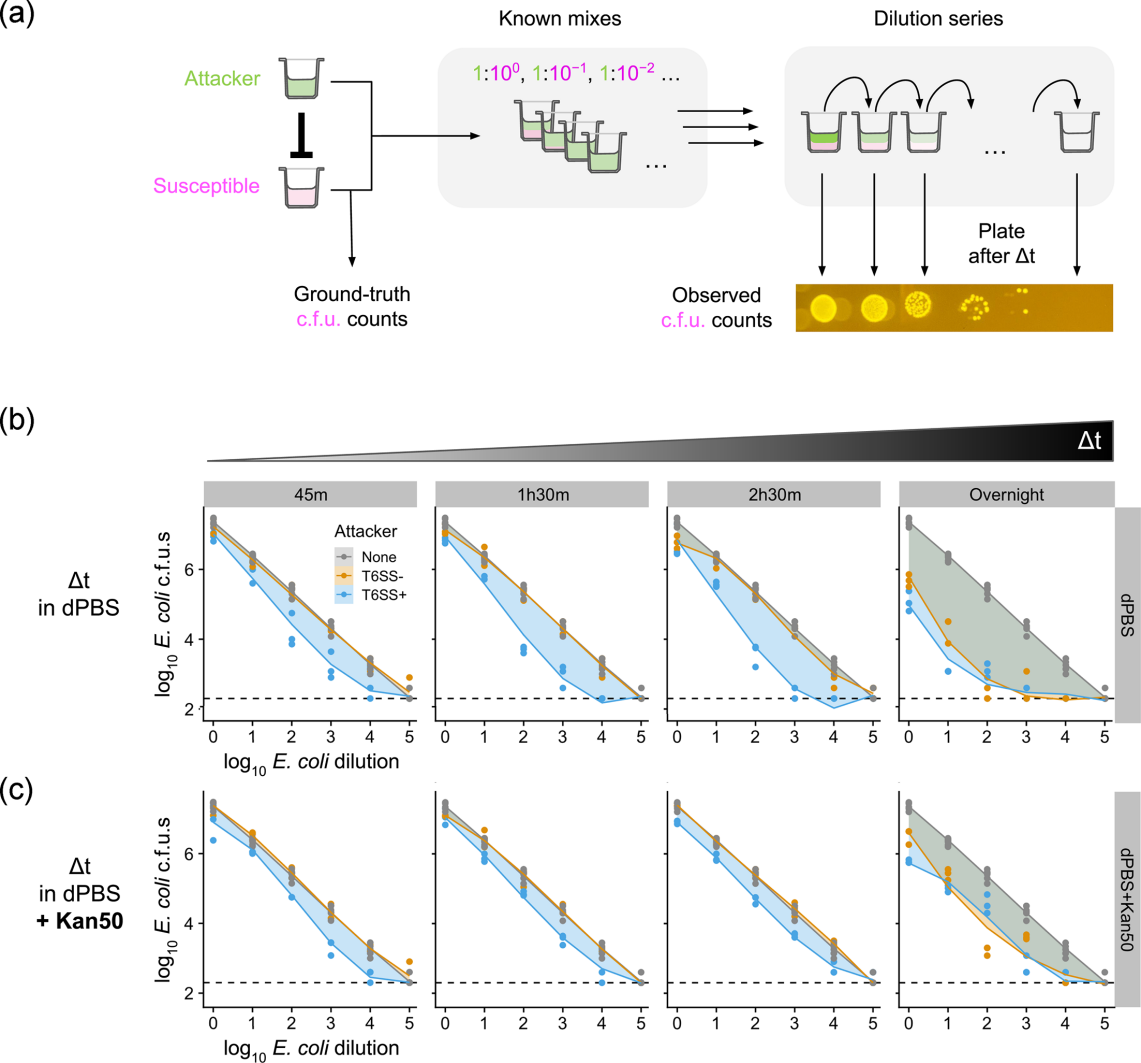
T6SS killing on selective media creates density-dependent bias in competition assays. (**a**) Schematic of ground-truth experiment, showing the preparation of different *A. baylyi*/*E. coli* mixtures to simulate possible outputs of a T6SS killing assay. After creation, mixtures are serially diluted and then incubated at room temperature for a variable time interval Δt, in the absence/presence of kanamycin before plating on selective media for c.f.u. counting. (**b**) Measurements of apparent *E. coli* recovery (c.f.u. per millilitre) in co-culture with T6SS− (Δhcp-*gfp*, orange) or T6SS+ (WT-*gfp*, blue) *A. baylyi* attackers, compared with known c.f.u. values determined from monocultures (‘none’, grey, replotted on each panel). Shaded areas highlight discrepancies between T6SS+/− treatments and the ‘true’ *E. coli* density; panels correspond to increasing incubation times Δt (incubation in dPBS only). Circles show individual datapoints; lines correspond to linear mixed effect model (fitting: maximum log likelihood, see Methods). Dashed lines show the detection limit (200 c.f.u. ml^−1^). (**c**) As (**b**), but for the same mixtures incubated in dPBS+kanamycin (50 µg ml^−1^) antibiotic pretreatment during Δt. *N*=3 pseudobiological replicates (independent platings of the same mixture) per condition (*N*=6 for ground-truth c.f.u. measurements).

To test whether this effect could bias the apparent outcome of a killing assay, we created a series of *A. baylyi*/*E. coli* mixtures, with fixed *A. baylyi* density and variable *E. coli* density. Shown in Fig. 2(a), these mixtures simulate the possible outputs of a typical killing assay, with susceptible cell survival varying from maximal (highest *E. coli* density, ~2.4×10^7^ c.f.u. ml^−1^) [7] down to the assay’s detection limit (lowest *E. coli* density, ~200 c.f.u. ml^−1^). Crucially, these mixtures are of known composition, enabling comparison of measured c.f.u. counts with known ‘ground-truth’ values. Fig. 2(b) compares ground-truth c.f.u. measurements (before mixing with *A. baylyi*) with observed *E. coli* c.f.u. values (after mixing with *A. baylyi*) for both T6SS+ and T6SS− mixtures (raw c.f.u. counts plotted in Fig. S1). Following 45 min of incubation in dPBS buffer, *E. coli* c.f.u. values were significantly reduced compared with ground-truth counts in mixtures with T6SS+ attackers (*P*=0.02, ANOVA with linear mixed effects model, see Methods). Shown in Fig. S2, the magnitude of this deviation (log ratio of observed/expected c.f.u. values) varied with *E. coli* density, with intermediate densities leading to the greatest discrepancies. We also confirmed that discrepancies disappeared in controls where T6SS was inactivated (*A. baylyi Δhcp-gfp*, *P*=0.34), demonstrating that these depletion effects arise via T6SS activity. Further, increasing the incubation period to 1.5 or 2.5 h progressively exacerbated discrepancies (Figs 2b and S2), suggesting that (i) T6SS killing also occurs while cells are suspended in dPBS buffer, and/or (ii) preincubation enhances residual killing on solid selective media. In contrast, when cell mixtures were left overnight (~18 h) before plating, we saw substantially reduced *E. coli* survival in *both* T6SS+ and T6SS− mixtures, suggesting that long-term incubation in dPBS reduces *E. coli* viability independently of T6SS activity. Overall, these results show that residual T6SS activity on selective media can lead to time- and density-dependent errors in apparent killing.

### Preincubation with antibiotics reduces bias arising from post-selection killing

While concerning, these results also suggested a potential intervention to mitigate viability discrepancies associated with T6SS killing. Bacterial cell suspensions are typically non-conducive to T6SS killing because they do not allow for prolonged cell–cell contact [16]. We therefore reasoned that, if our *A. baylyi+E. coli* cell suspensions were incubated with anti-*A. baylyi* antibiotics before plating, this would help to remove *A. baylyi* and thus prevent the T6SS killing we identified on solid selective media. To test this hypothesis, we repeated our assay, this time incubating cell mixtures in dPBS+50 µg ml^−1^ kanamycin, before plating on selective agar as before. While insufficient to completely remove discrepancies, this intervention was partially successful (i) in reducing the overall discrepancy between T6SS+ and T6SS− treatments (particularly for longer incubation periods) and (ii) in smoothing the density dependence such that all *E. coli* densities showed a similar (minor) deviation from ground-truth values (Fig. S2). We also showed that this antibiotic pre-treatment indeed reduces *A. baylyi* viability in a time-dependent manner (Fig. S1). In sum, these results show that antibiotic exposure prior to selective plating can mitigate viability discrepancies.

## Discussion

Competition assays are widely used to analyse microbial interactions [3,4, 7, 17,26]. The outcome of these assays is often evaluated using selective plating and c.f.u. counting, allowing co-cultured strains to be distinguished and enumerated based on their selective markers [27]. These techniques offer a simple and cost-effective way to quantify a focal strain’s growth or survival in the presence of other micro-organisms. C.f.u. counting also affords a very high dynamic range (typically >8 orders of magnitude), enabling viable cell counts from samples with very high or very low cell densities [28]. In studies aimed at estimating the anti-competitor effects of microbial weapons, such as T6SSs, c.f.u. counting has accordingly been the gold-standard evaluation method for decades [27,29].

Here, we show that T6SS-mediated killing continues even when cell mixtures are plated on selective media intended to disable attackers (Fig. 1). We also demonstrate that this effect leads to a significant and density-dependent bias in the apparent survival of the targeted strain (Figs 2 and S2). The bias increases with attacker/susceptible ratio, before falling as the *E. coli* density approaches the detection limit. This behaviour is consistent with the contact-dependent nature of T6SSs. The fewer the surviving *E. coli* bacteria following a competition assay, the lower the dilution at which their c.f.u. values are counted, and so the higher the attacker density present within the corresponding c.f.u. spot. Higher attacker cell densities lead to more attacker-susceptible cell contact [13,30] and so more T6SS killing on selective media, decreasing the apparent *E. coli* survival.

Importantly, this coupling between post-competition susceptible survival and attacker density in the c.f.u. dilution series means that residual killing on selective media does more than simply extend the effective competition period – it amplifies apparent killing in situations where *E. coli* survival is already low. Residual killing on selective media thus has the potential to introduce large (>10-fold) errors to the quantification of competition outcome. Specifically, the effects of high-potency weapons and toxins could be *overestimated*, since (i) competition is extended beyond the intended interaction time and (ii) transferring cells to selective media generally involves resuspension and re-assortment, further increasing killing [30]. For the same reasons, *resistance* to weapon attacks could be *underestimated*, since residual killing can significantly reduce apparent survival of the target strain, post-competition [4,25, 31].

Our findings have several limitations. First, while *A. baylyi*/*E. coli* cocultures are an established model for understanding T6SS interactions [3,7, 10,13], this is but one example of a lethal microbe–microbe interaction. Other attacker-target pairings may introduce additional factors that modulate intermicrobial competition (e.g. differential adhesion [20,32] and density-dependent toxin production [33,34]), potentially introducing other biases alongside those shown here. Our findings are also limited to a specific type of selection (kanamycin); other selective media may result in different residual killing profiles. We speculate that bacteriostatic, colourimetric or non-auxotrophic media may be particularly vulnerable to residual killing biases, since these would be expected to disable attacker strains less rapidly (or not at all) compared with media containing bacteriolytic antibiotics. Conversely, it is possible that some c.f.u. counting methods may be intrinsically less prone to bias than others. The recently published geometric volumetric analysis method [28] counts c.f.u. values by embedding cells in agarose, rather than growing them on top of it, and so may mitigate biases by physically separating attacker and target cells. Further work is required to test these possibilities.

Immediately, however, our findings emphasize the need for caution when interpreting the results of co-culture c.f.u. counts, especially when lethal microbe–microbe interactions are (or may be) involved. We speculate that our findings could extrapolate to other rapid killing mechanisms, including other contact weapons [32,34] and diffusible toxins [35,36], with biases varying according to each weapon’s potency, density dependence and regulation in response to antibiotic stress. Based on our findings, we recommend that, where co-cultured microbes are known to interact (particularly via potent antimicrobial toxins), researchers perform ground-truth tests to check for biases. Where biases are present, we showed that these may be partially alleviated by incubating cell suspensions with antibiotics before plating. We note that some bacteria (e.g. *Vibrio cholerae* C6706 [30]) suppress T6SS activity at low temperatures, and so another intervention, which we did not test here, might be to cool mixed cell suspensions before c.f.u. plating. Failing this, alternative measures of competition outcome where cells are physically separated (e.g. flow cytometers [37] and qPCR [38]) or effectively enumerated *in situ* (barcode sequencing [39], quantitative fluorescence microscopy [13,40] or non-selective colourimetric killing assays [3,41, 42]) may become preferable.

C.f.u. counting has many strengths and is likely to remain a common technique for quantifying the outcomes of competition assays. However, our work warns against uncritical use of this method, as residual killing effects can lead to misleading results where competition involves fast-killing antimicrobials.

## Supplementary material

10.1099/mic.0.001600 Uncited Supplementary Material 1.

## Abbreviations

c.f.u.: colony forming unit
T6SS: type VI secretion system
LB: Lysogeny Broth
dPBS: Dulbecco’s PBS
EGFP: Enhanced Green Fluorescent Protein
