# Supplementary material for: Type VI secretion system activity at lethal antibiotic concentrations leads to overestimation of weapon potency
Source: Microbiology (Reading). 2025 Aug 21;171(8):001600. doi: 10.1099/mic.0.001600 (PMC12370439; doi:10.1099/mic.0.001600)
Supplement: Uncited Supplementary Material 1. [file mic-171-01600-s001.pdf]

Supplementary Information

Type VI secretion system activity at lethal antibiotic concentrations leads to overestimation of weapon potency

Authors: William P. J. Smith *,[1,†], Elisa T. Granato *,[2,†]

*Corresponding authors:
william.smith-4@manchester.ac.uk,
elisa.granato@gmail.com.
[†]Equal author contribution.

Supplementary Figures

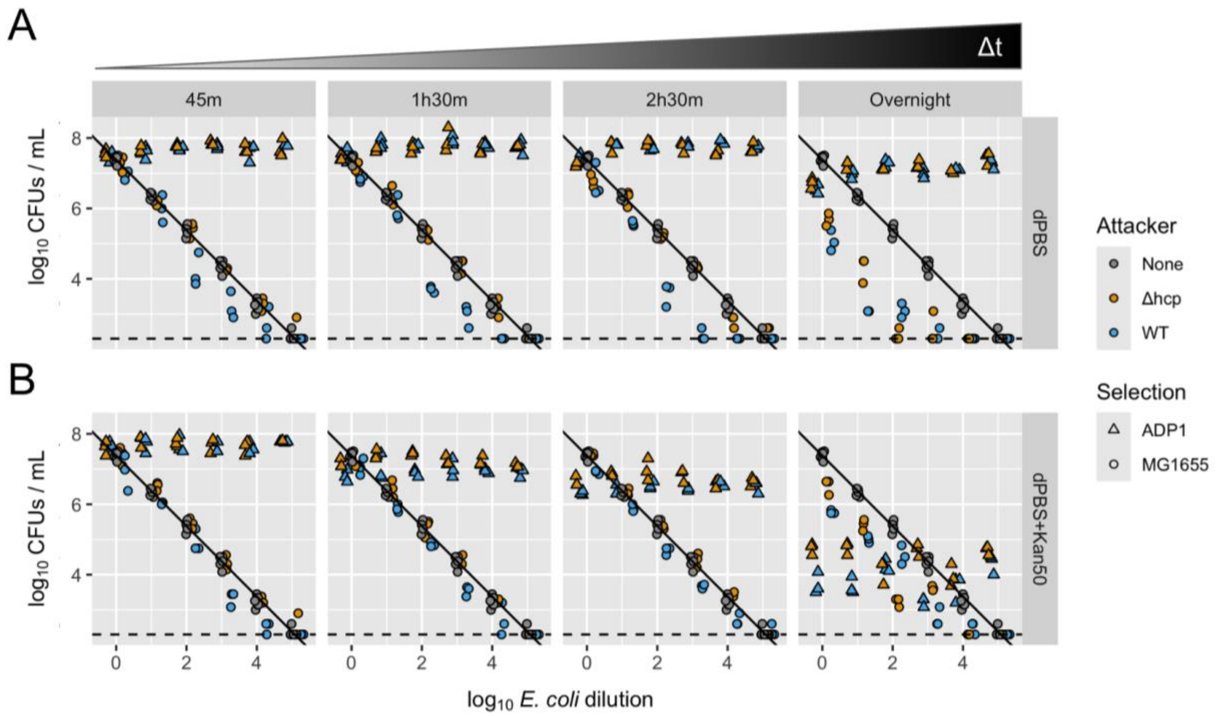

Fig. S1: Raw data for "Ground truth" experiment shown in Fig 2. (A) Measurements of apparent *E. coli* ("MG1655", circles) and *A. baylyi* ("ADP1", triangles) recovery are coloured according to attacker type ("Δhcp": T6SS−; "WT", T6SS+), and plotted alongside known *E. coli* CFUs determined from monocultures ("None", grey, replotted on each panel). Panels correspond to increasing incubation times Δt. Dashed lines show the detection limit (200 CFU/mL); solid line shows the predicted *E. coli* recovery (CFU = Dilution) based on extrapolation from the undiluted *E. coli* sample (2.4 x 10^7 CFU/mL). **(B)** As (A) but for same mixtures incubated in dPBS + kanamycin (50 µg/mL) antibiotic pretreatment during Δt. N = 3 pseudobiological replicates (independent platings of the same mixture) per condition (N = 6 for ground truth CFU measurements).

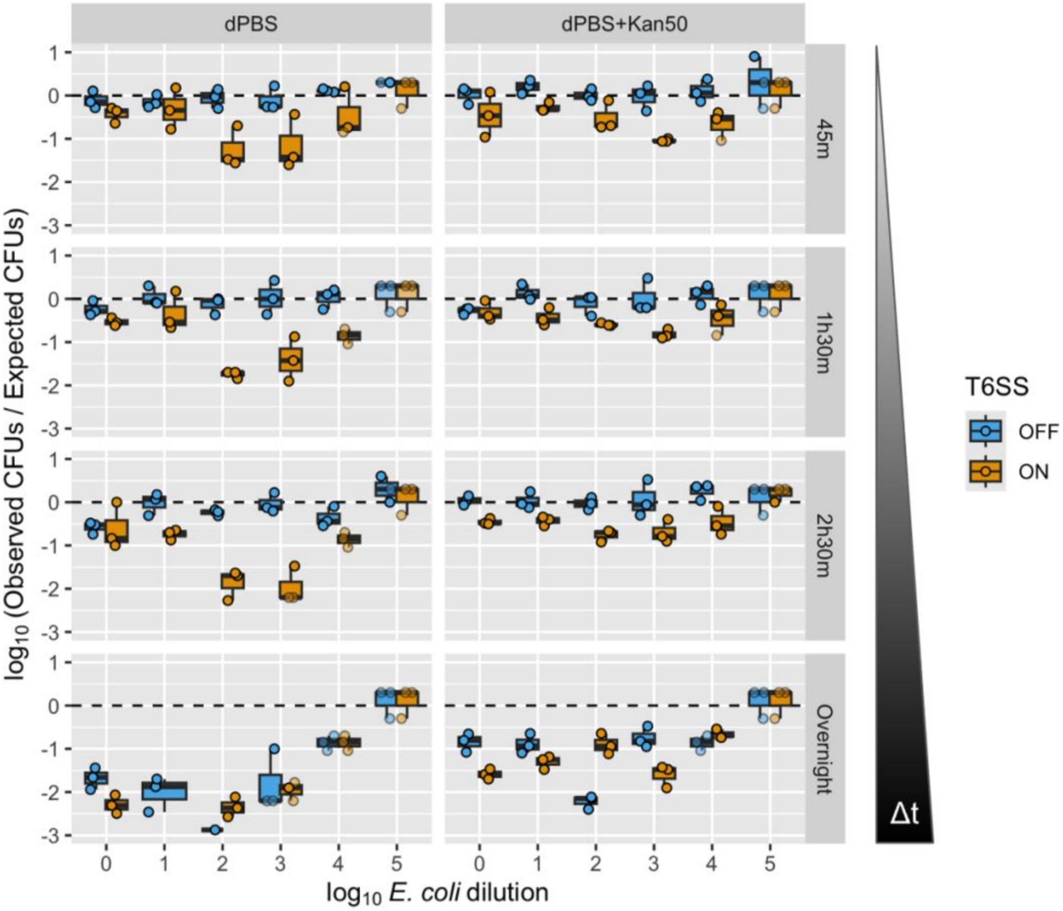

Fig. S2: Discrepancy effects sizes for "Ground truth" experiment shown in Fig 2. Here we replot the data shown in Figs. 2B and S1 to highlight the magnitude of discrepancies between *E. coli* CFUs recovered following serial dilution of *A. baylyi / E. coli* mixtures ("Observed CFUs"), and those CFUs determined from *E. coli* monocultures before mixing ("Expected CFUs"). Dashed lines correspond to Observed CFUs = Expected CFUs. T6SS "OFF" and "ON" (legend) correspond to *A. baylyi* Δhcp and WT treatments; columns compare pretreatments without / with Kanamycin (50 μg/mL) during Δt. Semi-transparent datapoints mark CFU ratios where observed survival falls below the detection limit (200 CFU/mL), leading to underestimation of discrepancy. For overnight incubations, *E. coli* recovery is less than expected even in T6SS "OFF" treatments, showing that in this case biases result from processes other than residual T6SS killing. N = 3 pseudobiological replicates (independent platings of the same mixture) per condition.
